# Lack of Evidence for Kynurenine Pathway Dysfunction in Huntington’s Disease: CSF and Plasma Analyses from the HDClarity Study

**DOI:** 10.1177/18796397241301761

**Published:** 2024-12-10

**Authors:** Vinod Khetarpal, Todd Herbst, Celia Dominguez, Ignacio Munoz-Sanjuan, Cristina Sampaio, Bryan Marks, Dennis L. Miller, James Farnham, Aaron Ledvina, Hannah Anglehart, Imran Rehmani, Amber LaFayette, Natasha Spridco, Douglas Langbehn, Edward J. Wild, Robert Pacifici

**Affiliations:** a CHDI Management, Inc., the company that manages the scientific activities of CHDI Foundation, Inc., 155 Village Boulevard, Princeton, NJ, USA; b LabCorp, Early Development Laboratories Inc. 3402 Kinsman Blvd, Madison, WI, USA; c Carver College of Medicine, University of Iowa, Iowa City, IA, USA; d University College London Huntington’s Disease Centre, UCL Queen Square Institute of Neurology, London, UK

**Keywords:** Kynurenine, 3-hydroxy-kynurenic acid, kynurenic acid, anthranilic acid, quinolinic acid, tryptophan, CSF, plasma, Huntington’s disease, biomarkers

## Abstract

**Background:**

Evidence from animal studies and post-mortem studies of brains from people with Huntington’s disease (PwHD) has suggested that the kynurenine pathway (KP) may be dysregulated in Huntington’s disease (HD).

**Objective:**

To determine whether there are differences in KP metabolites in the CSF and plasma of PwHD versus healthy controls enrolled in the HDClarity study.

**Methods:**

CSF and plasma samples from 141 PwHD with mild and moderate manifest disease and 75 healthy controls were analyzed for 3-hydroxykynurenine (3-OH-KYN), quinolinic acid, kynurenine, anthranilic acid, kynurenic acid, and tryptophan concentrations using validated high-performance liquid chromatography with tandem mass spectrometry (LC-MS/MS) methods. The primary and secondary endpoints compared metabolite concentrations between groups, and an exploratory analysis (PwHD only) evaluated the association between the metabolite levels and severity of disease.

**Results:**

No significant differences in CSF or plasma concentrations of any of the six KP metabolites were observed between PwHD and controls, and there were no strong associations between the concentration of any KP metabolite and disease severity. A principal component analysis of the combined CSF and plasma measures showed a substantial positive correlation among all metabolites except for tryptophan in plasma.

**Conclusions:**

We found no evidence to support the hypothesis of dysregulation of KP metabolites in HD based on CSF and plasma metabolite levels. The monitoring of KP metabolites in CSF or plasma is unlikely to serve as a pharmacodynamic biomarker for disease progression or therapeutic intervention in HD.

## Introduction

**Figure 1:**
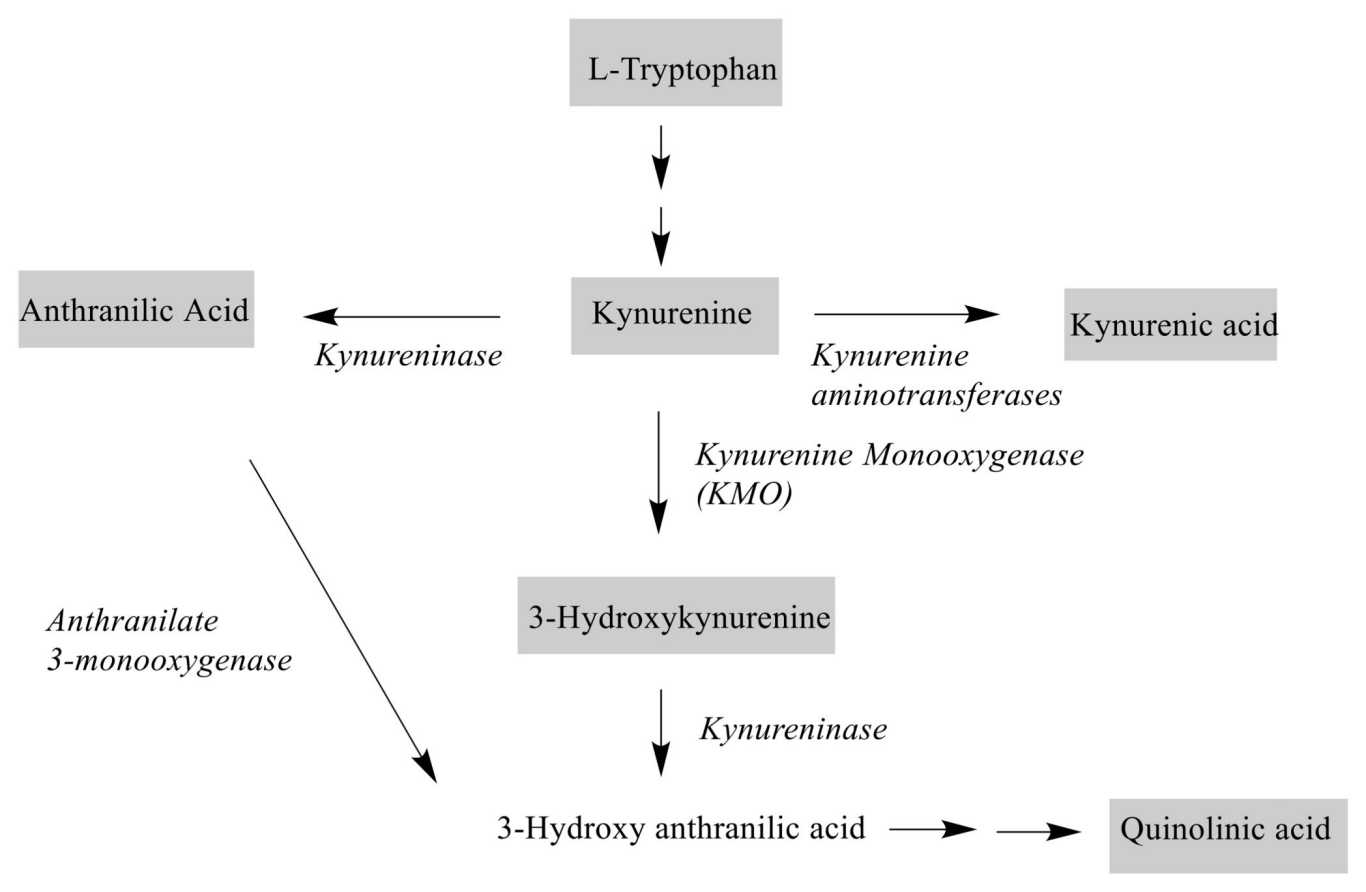
Kynurenine metabolic pathway showing the metabolites (in grey boxes) quantitated in CSF and plasma.

The kynurenine pathway (KP) (Figure 1) is the major metabolic pathway for the essential amino acid tryptophan and plays a critical role in generating cellular energy via the synthesis of nicotinamide adenine dinucleotide. Dysfunction of this pathway has been postulated to contribute to the pathophysiology of neurodegenerative diseases, including Huntington’s disease (HD).^1^ The KP metabolites have various neuronal effects, they could either be neuroprotective or neurotoxic. Two downstream metabolites of kynurenine, 3-hydroxy-kynurenine (3-OH-KYN) and quinolinic acid have been associated with neurodegeneration^2,3^ and their levels have been reported to be increased in the postmortem brains of PwHD.^4^ 3-OH-KYN has been reported to generate reactive oxygen species that cause neurodegeneration.^5^ Injections of quinolinic acid have long been known to induce excitotoxic lesions via activation of N-methyl-D-aspartate (NMDA) receptors.^6^ When injected into the striatum of rodents and primates, it causes selective loss of the medium spiny neurons while sparing several other intraneuronal cell populations.^7–9^ On the other hand, kynurenic acid has been shown to be neuroprotective.^10^ It acts as an antagonist for NMDA receptors and therefore blocks neurotoxic effects of quinolinic acid.^11^

The role of KP metabolites in HD is important to understand as it could potentially provide a biological rationale to support modulation of KP metabolism as a therapeutic strategy for HD^12^, as well as the development of KP metabolites as pharmacodynamic biomarkers. However, previous studies investigating KP metabolites in PwHD^13–15^ were either underpowered or utilized non-validated analytical methods. Sensitive and selective LC-MS/MS methods have now been validated according to FDA guidelines^16^ for six KP metabolites (kynurenine, 3-OH-KYN, kynurenic acid, quinolinic acid, anthranilic acid, and tryptophan) in human cerebrospinal fluid (CSF) and plasma.^17^ Using these methods, we aimed to determine whether there are differences in KP metabolites in the CSF and plasma of PwHD versus healthy controls.

## Materials and Methods

### Procurement of Samples

Plasma and CSF samples were obtained from the ongoing HDClarity study (NCT02855476), which is a multi-site CSF collection initiative to facilitate therapeutic development for HD.^18^ The study is designed to collect biofluid samples from PwHD across the spectrum of the disease, from early pre-manifest to advanced manifest HD as well as healthy controls. Sample collection methods were optimized for analysis of metabolites with a 6-hour fast before sampling, CSF cooled on ice at the point of collection and sample processing beginning within 15 minutes of collection. Participant medical history and clinical measures are collected through the Enroll-HD clinical platform.^19^ Both HDClarity and Enroll-HD are conducted in accordance with the Declaration of Helsinki; all patients provide written informed consent for participation for future analyses based on their data. The HDClarity study was approved centrally by Camberwell St Giles Research Ethics Committee (IRAS 185506) and each site’s ethics board.

For the purposes of our study, samples were procured from healthy controls and PwHD with a UHDRS Diagnostic confidence score = 4; CAG expansion ≥ 36 and UHDRS Total Functional Capacity (TFC) scores between 4 and 13. Exclusion criteria followed those already outlined in HDClarity protocol including screening test results for C-reactive protein levels >2x the upper limit of normal.

A subset of blinded HDClarity samples were obtained in two batches from the sample repository (BioRep srl, Milano, Italy); the first batch of samples from 72 participants and a second batch of samples from 144 participants were shipped frozen in dry ice to the bioanalytical laboratory and were analyzed for KP metabolites within 3 months of receipt. Samples were analyzed in a blinded manner; participant characteristics were only available to the individual who performed the statistical analysis (DL).

### Sample Analysis

Quantitation of kynurenine, kynurenic acid, 3-OH-KYN, anthranilic acid, quinolinic acid and tryptophan in CSF and plasma were conducted at the central bioanalytical laboratory (LabCorp, Madison, WI) using methods as previously described by Khetarpal et al^17^ and summarized in the **supplementary appendix**. Briefly, three LC-MS/MS methods were validated for each of the two matrices, CSF, and plasma: a 4-analyte assay (simultaneous determination of kynurenine, kynurenic acid, anthranilic acid, and 3-OH-KYN), an assay for quinolinic acid, and an assay for tryptophan. The dynamic range and limits of quantitation were based on concentrations observed endogenously for each analyte. We used isotopically labeled internal standards for all assays.

### Statistical Analysis Plan

The sample size was estimated using preliminary data from a pilot study that determined the levels of KP metabolites in 20 PwHD with manifest disease and 14 healthy controls. Effect size estimates were generated for the three primary metabolites, 3-OH-KYN, kynurenine, and quinolinic acid utilizing two-sided hypothesis testing.

The primary hypothesis was that there would be differences between PwHD and controls in CSF concentrations of 3-OH-KYN, kynurenine, and quinolinic acid. Between group differences in KP metabolite concentrations were analyzed using analysis of covariance (ANCOVA) models including age, age-by-HD status, and sex as covariates. As anticipated from the pilot data, the metabolite concentration distributions were substantially skewed (Figure e1), and we used natural log transformations for statistical modeling and inference. The overall analysis can be viewed as a covariate-adjusted t-test with possible unequal variances between the groups being compared. Statistical significance was defined as a Bonferroni-corrected 5% family-wise error rate among these 3 metabolites. This translates to an uncorrected p-value threshold of 0.0167.

Secondary objectives were to determine whether there are between group differences in the CSF concentrations of kynurenic acid, tryptophan, and anthranilic acid and in plasma concentrations of all 6 KP metabolites. Exploratory analyses looked for between group differences in the following ratios (metabolite log values): 3-OH-KYN/kynurenine; 3-OH-KYN/kynurenic acid; kynurenic acid/kynurenine and quinolinic acid/kynurenine. Secondary and exploratory analyses were performed using ANCOVA models as described for the primary analysis.

Correlations between KP metabolite levels in plasma and CSF were calculated via the Pearson correlation coefficients of the non-transformed and log values, as well as Spearman Rank correlations. Correlation analyses concluded with Principal Component Analyses (PCA) of these correlation matrices to assess the essential dimensionality of the metabolite data.

Finally, we explored the relationships of KP metabolite levels with various clinical measures (Unified Huntington’s Disease Rating Scale [UHDRS] motor score, UHDRS total functional capacity [TFC], symbol digit modality test, Stroop word-reading test, Stroop color-naming test, and composite UHDRS score) as captured in the Enroll-HD platform. These analyses were limited to only PwHD. Clinical measures were used as outcomes in linear regressions, with log concentrations of each metabolite as the predictor. All analyses were based on log transformations of metabolite levels and controlled for age, sex, and educational level (ISCED) and were performed with and without adjustment for multiplicity. We also fit a linear model to test relationships between metabolite levels by disease burden statistic, CAP30 (age*[CAG-length-30])^20^ within the HD group. To test the adequacy of CAP30 for describing possible metabolite relationships to CAG length and age, we also fit linear models that explicitly uses age, CAG length, and their interaction as predictors.

## Results

CSF and plasma samples were obtained from 216 HDClarity participants (PwHD, n=141; Control, n=75). Among the HD group, 15 participants were categorized as having moderate disease and the remaining 126 had early HD. The mean ±SD age for PwHD was 53.2 ±10.1 years and 50.13 ±12.0 years in the control group; age was not significantly different between groups (p=0.061). The HD group comprised 81 (57.4%) male and 60 (42.6%) female participants, and the control group included 33 (44.0%) male and 42 (56.0%) female participants (p=0.082).

**Figure 2:**
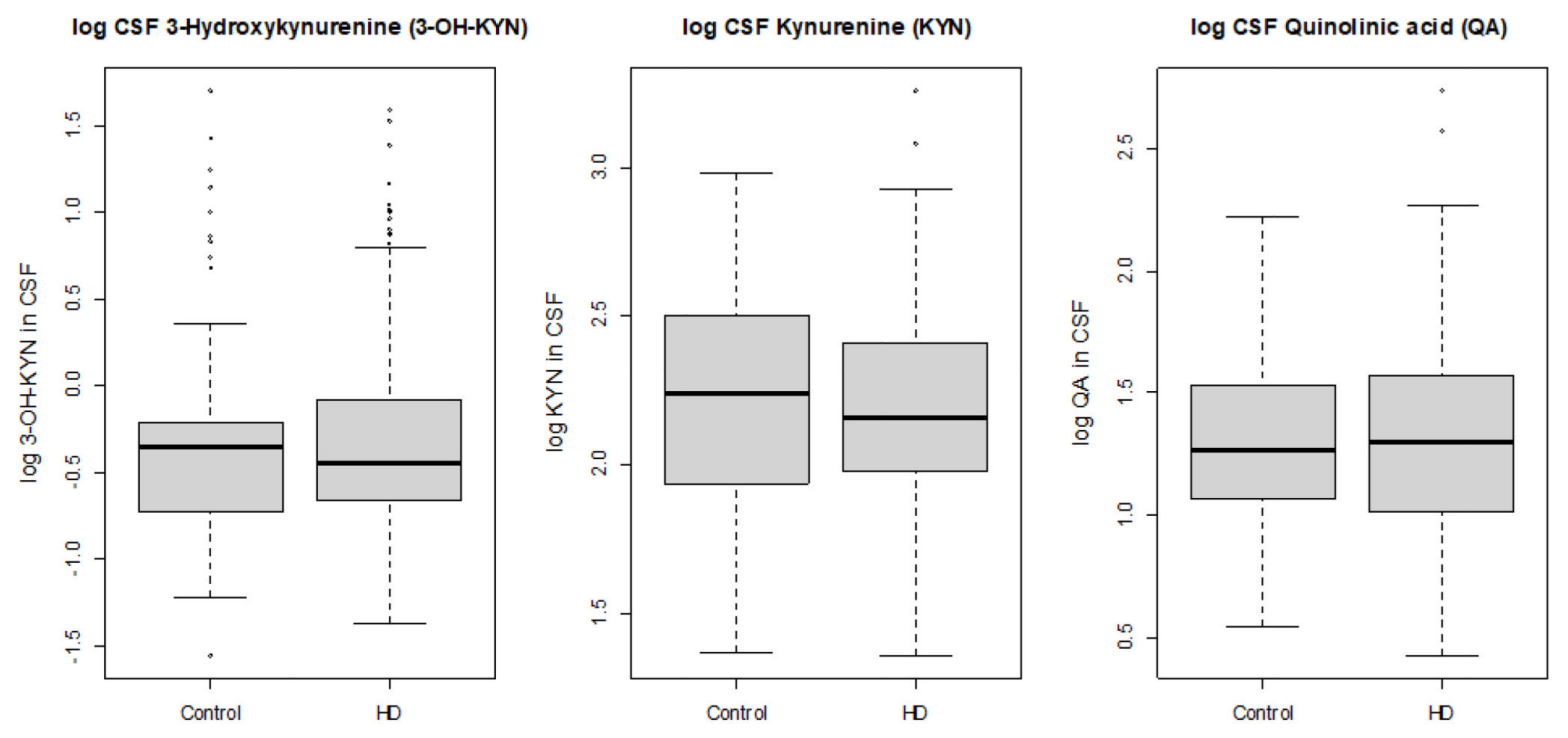
Unadjusted boxplots of log-transformed metabolite concentrations (ng/mL) in control and PwHD.

There was no evidence of differences in CSF concentrations of 3-OH-KYN, kynurenine, and quinolinic acid between PwHD and control participants (Table 1, Figure 2). An incidental finding was a strong relationship between increasing levels of all three metabolites with increasing age (Figure e2, Table e1). For quinolinic acid, the CSF concentrations were higher in males than in females (p=0.03). An additional age-by-sex interaction was tested in all three metabolite models and did not show significance in any of them.

Similarly, we found no evidence of significant differences between PwHD and control participants (Table 1, Figures e3 and e4) in: 1. CSF concentrations of anthranilic acid, kynurenic acid, and tryptophan; 2. plasma concentrations of any of the six KP metabolites; 3. CSF ratios of 3-OH-KYN/kynurenine, 3-OH-KYN/kynurenic acid, kynurenic acid /kynurenine, and quinolinic acid/kynurenine.

**Figure 3:**
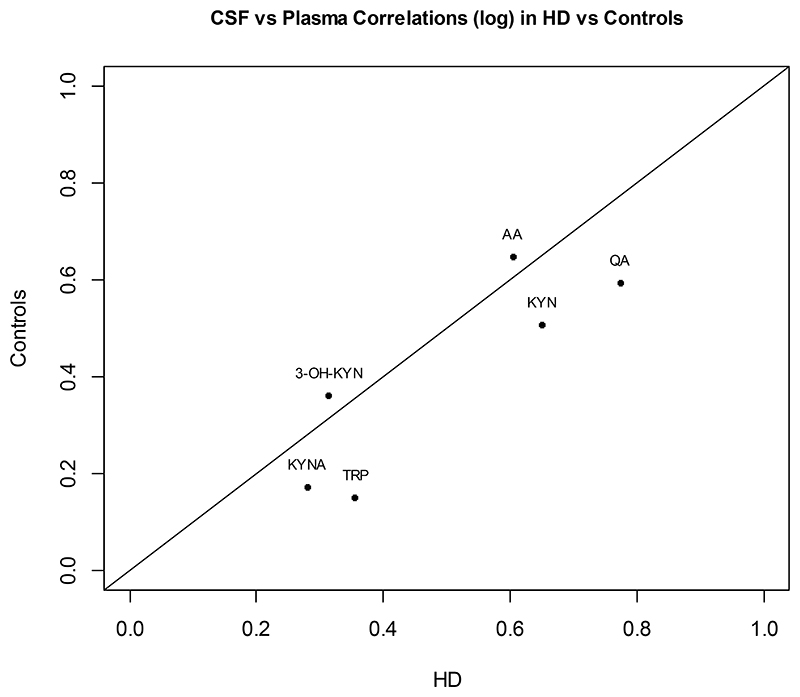
Plasma and CSF correlations of log transformed concentrations (ng/mL) of the six metabolites in people with HD (PwHD) versus controls. Abbreviations: 3-OH-KYN, 3-hydroxykynurenine; AA, anthranilic acid; HD, Huntington’s disease; KYN, kynurenine; KYNA, kynurenic acid; QA, quinolinic acid; TRP, tryptophan.

Correlations between plasma and CSF concentrations were relatively high for kynurenine, quinolinic acid, and anthranilic acid, whereas the correlations were lower but still highly significant for the other three metabolites (Figure 3, Table 2). A PCA of the combined CSF and plasma measures showed a substantial positive correlation among all metabolites except for tryptophan in plasma. The PCA of the log values of plasma and CSF metabolite levels in the combined HD and control groups yielded the first 6 principal component correlation values as shown in Table 3. These six components accounted for 0.850 of the total variation. Only the first three components appeared to be related (r >0.40) to more than a single metabolite; PC1 accounted for 0.399 of the data variances, PC2 for an additional 0.116, and PC3 for an additional 0.100 of the variance. Notably, there were no apparent associations between any of these principal components and HD status (Table e2).

Finally, we explored possible associations of CSF and plasma KP metabolite levels with various clinical measures in PwHD. Based on false discovery rates (*fdr*), there was little evidence of relationships between any of the CSF or plasma KP metabolite levels and clinical measures of HD severity as assessed by UHDRS motor score, UHDRS TFC, symbol digit modality test, Stroop word-reading test, Stroop color-naming test, and composite UHDRS score (Tables e3 and e4). The only potentially relevant associations (with a *fdr* <0.15) showed that lower CSF kynurenine levels were associated with poor Symbol Digit scores (*fdr*=0.085), and that higher CSF 3-OH-KYN (*fdr* = 0.078) and plasma tryptophan (*fdr* = 0.126) concentrations were associated with worse disease burden as assessed by CAP30 scores (Table e5).

The disease burden formula is a product of age. To control for the potential age effect, we refit the models by replacing CAP30 score with explicit predictor terms for age, CAG length, and age-by-CAG interaction; data are shown in Table e6. Among CSF metabolites, there was some evidence of specific associations between the age-by-CAG interaction and 3-OH-KYN, kynurenine, and tryptophan (all *fdr* = 0.152). Testing the joint significance of the main CAG effect and age-by-CAG interaction, the association was strengthened for 3-OH-KYN and tryptophan, with *fdr* = 0.119 for both metabolites. Among plasma metabolites, there was an association between tryptophan and the age-by-CAG interaction (*fdr* = 0.029) that was larger than the tryptophan CAP30 association, suggesting that the CAP30 association is not simply due to age differences.

## Discussion

We undertook this study to obtain definitive data on the levels of KP metabolites in CSF and plasma of PwHD and compare these to the levels in healthy controls using validated quantification methods. Based on CSF and plasma metabolite levels, we found no evidence to support the hypothesis of dysregulation of KP metabolites in HD.

Our findings confirm and extend the recently published finding by Rodrigues et al.^15^, who found, albeit in a much smaller study, that KP metabolites in CSF and plasma were stable over 6 weeks and that there were no significant differences between healthy controls, PwHD with premanifest disease, and PwHD with manifest disease. However, as in our study, CSF concentrations of kynurenic acid, 3-OH-KYN, and quinolinic acid did increase significantly with age. Whereas ‘upstream’ metabolites such as kynurenine and kynurenic acid have been described as neuroprotective^11^, the ‘downstream’ metabolites 3-OH-KYN and quinolinic acid were reported as neurotoxic^2,3^, and we therefore explored various ratios of metabolites within CSF or plasma to provide potential insights into any changes within the metabolic pathways. The ratios (in both CSF and plasma) of 3-OH-KYN to kynurenine, 3-OH-KYN to kynurenic acid, kynurenic acid to kynurenine, and quinolinic acid to kynurenine showed no significant differences between HD and healthy control groups, indicating no notable shift in these metabolic pathways due to HD.

We also explored the correlation of KP metabolite levels in plasma and CSF within individuals and found that all metabolites showed significant correlations between the two matrices, whether PwHD and control groups were considered separately or together as one set. Similar results for correlations of KP metabolites in serum vs. CSF were reported by Sovang et al.^21^ who also showed age-related increase in CSF levels of kynurenine and quinolinic acid. There is no clear explanation for the higher correlations between plasma and CSF concentrations of kynurenine and quinolinic acid compared to other metabolites. Whereas kynurenine is highly permeable and can readily cross the blood-brain barrier, quinolinic acid has low permeability and has limited uptake into the brain. Other factors—such as protein binding, active transport across blood-brain and blood-CSF barriers, and enzymatic processes for formation and degradation in the central and peripheral compartments—may be more relevant in determining the relationship between peripheral and CSF levels of KP metabolites. In the principal component analysis of the CSF and plasma concentrations, the primary component reflected high mutual correlation among 8 of the 12 metabolites, moderate correlation for 3, and low correlation for one (tryptophan in plasma). These results suggest a low essential dimensionality of the combined KP metabolite data in CSF and plasma. No association was found between any of the principal components and HD status.

Although a few possible statistically significant associations (lower CSF kynurenine concentrations were associated with poor symbol digital scores, and lower plasma kynurenine and kynurenic acid concentrations were associated with poor clinical outcome measures) were apparent before adjusting for multiplicity, there was no convincing association between any of the metabolite levels and any of the clinical measures of HD severity after false discovery rate adjustment for multiple comparisons. However, our analyses did suggest some association between KP metabolites (e.g. CSF concentrations of 3-OH-KYN, kynurenine, and tryptophan) and HD disease burden as defined by age and CAG length. The pathological significance of these results is unclear but may point to minor changes in KP metabolic pathway in HD. Nonetheless, these minor changes were not clearly reflected in differences in clinical status.

In summary, our comparison of KP metabolite levels in CSF and plasma between PwHD and healthy controls did not provide evidence in support of the hypothesis of clinically relevant KP dysregulation in HD. While we cannot exclude localized KP alterations in most-affected brain regions, whose signal is diluted when examining CSF or plasma, based on the results obtained in this study, it is difficult to support targeting the KP pathway (e.g., with kynurenine monooxygenase inhibitor, CHDI-340246)^12^ as a therapy for HD. Furthermore, the absence of a strong relationship between the level of any of the KP metabolites in either CSF or plasma with HD clinical measures or disease burden based on CAG length suggests that KP metabolite levels cannot be used as pharmacodynamic biomarkers either during therapeutic intervention or disease progression.

## Supplementary Material

Supplementary Materials

## Figures and Tables

**Table 1:** Unadjusted group comparisons (PwHD vs healthy controls) of log concentrations (ng/mL) of kynurenine pathway metabolites.

Metabolite	Log Concentration Difference (SE)	t value	p value
**Primary metabolites of interest in the CSF**			
3-OH-KYN	-0.002 (0.089)	-0.02	0.98
Kynurenine	-0.033 (0.048)	-0.68	0.50
Quinolinic acid	-0.049 (0.052)	-0.94	0.35
**Secondary metabolites of interest in the CSF**			
Anthranilic acid	-0.023 (0.064)	-0.37	0.72
Kynurenic acid	0.032 (0.067)	0.48	0.63
Trytophan	-0.028 (0.031)	-0.93	0.36
**Metabolites of interest in the plasma**			
3-OH-KYN	-0.038 (0.040)	-0.97	0.33
Kynurenine	-0.007 (0.033)	-0.20	0.84
Quinolinic acid	-0.009 (0.043)	-0.20	0.84
Anthranilic acid	0.006 (0.043)	0.14	0.89
Kynurenic acid	-0.071 (0.047)	-1.51	0.13
Trytophan	-0.033 (0.023)	-1.41	0.16
**Pathway ratios of interest in the CSF**			
3-OH-KYN/ kynurenine	0.031 (0.094)	0.33	0.74
3-OH-KYN/kynurenic acid	-0.026 (0.106)	-0.24	0.81
Kynurenic acid / kynurenine	0.031 (0.094)	0.33	0.74
Quinolinic acid/ kynurenine	-0.028 (0.050)	-0.56	0.58
**Pathway ratios of interest in the plasma**			
3-OH-KYN/ kynurenine	-0.032 (0.031)	-1.01	0.32
3-OH-KYN/ kynurenic acid	0.033 (0.043)	0.77	0.44
Kynurenic acid/ kynurenine	-0.032 (0.032)	-1.01	0.32
Quinolinic acid/ kynurenine	-0.002 (0.324)	-0.06	0.95

Abbreviations: 3-OH-KYN, 3-hydroxykynurenine

**Table 2:** Correlation of plasma and CSF metabolite levels (ng/mL).

	Combined PwHD and Controls			PwHD Only			Controls Only		
Metabolite	r	Spear r	r of log	r	Spear r	r of log	r	Spear r	r of log
3-OH-KYN	0.236	0.402	0.327	0.278	0.366	0.312	0.164	0.488	0.363
Kynurenine	0.620	0.577	0.601	0.671	0.600	0.648	0.491	0.520	0.508
Quinolinic acid	0.717	0.705	0.719	0.779	0.760	0.773	0.537	0.596	0.595
Anthranilic acid	0.302	0.581	0.619	0.273	0.594	0.605	0.665	0.564	0.649
Kynurenic acid	0.230	0.264	0.232	0.268	0.312	0.279	0.161	0.212	0.173
Tryptophan	0.282	0.267	0.270	0.369	0.333	0.354	0.151	0.161	0.151

r = Pearson correlation; Spearman r = Spearman Rank correlation; r of log = Pearson correlation of log transformed values. Abbreviations: 3-OH-KYN, 3-hydroxykynurenine; PwHD, people with Huntington’s disease.

**Table 3:** Principal component analysis of CSF and plasma metabolite log transformed levels.

Metabolite	PC1	PC2	PC3	PC4	PC5	PC6
**CSF metabolites**						
3-OH-KYN	0.445	-0.576	-0.421	0.099	0.260	0.103
Kynurenine	0.791	0.070	0.081	0.365	-0.242	-0.038
Quinolinic acid	0.754	-0.321	-0.430	0.021	0.039	0.042
Anthranilic acid	0.666	0.349	-0.147	-0.143	-0.391	-0.248
Kynurenic acid	0.465	-0.150	0.411	0.364	-0.355	0.542
Tryptophan	0.527	0.245	-0.146	0.646	0.041	-0.357
**Plasma metabolites**						
3-OH-KYN	0.662	-0.352	0.391	-0.140	0.189	-0.216
Kynurenine	0.844	0.143	0.130	-0.153	0.246	0.156
Quinolinic acid	0.787	-0.211	-0.138	-0.310	-0.046	-0.008
Anthranilic acid	0.594	0.367	-0.131	-0.462	-0.294	0.124
Kynurenic acid	0.500	0.046	0.614	-0.105	0.328	-0.177
Tryptophan	0.291	0.649	-0.197	0.102	0.566	0.298

Abbreviations: 3-OH-KYN, 3-hydroxykynurenine

## Data Availability

Full results of this analysis are provided in the Supplemental tables.
