# Supplementary material for: Lack of Evidence for Kynurenine Pathway Dysfunction in Huntington’s Disease: CSF and Plasma Analyses from the HDClarity Study
Source: J Huntingtons Dis. Author manuscript; Available in PMC 2025 Feb 3. (PMC7617368; doi:10.1177/18796397241301761)
Supplement: Supplementary Materials [file EMS202864-supplement-Supplementary_Materials.docx]

**Supplemental Information**

**Sample analysis**

For the 4-analyte assay in CSF and plasma, chromatographic separation of the analytes was achieved using a Waters XSelect HSS T3 analytical column (2.5 µm particle size, 2.1 x 50 mm; Waters Corp., Milford, MA) set at 30°C. A multistep gradient using mobile phase A comprised of EDTA (10 mM, aqueous):water:formic acid (0.05:100:0.1, v:v:v) and mobile phase B containing EDTA (10 mM, aqueous):acetonitrile:methanol:formic acid (0.05:50:50:0.1, v:v:v:v) was used for elution of analytes. MS analysis was performed using an API 5500 instrument in ESI+ mode with the following transitions monitored: m/z kynurenine, 209.2→146.1; m/z kynurenine-D_6_, 215.2→152.1; m/z; Kynurenic acid , 190.0→144.1; m/z Kynurenic acid -D_5_, 195.0→149.1; m/z anthranilic acid, 137.9→91.9; m/z anthranilic acid-^13^C_6_, 143.9→97.9; m/z 3-OH KYN, 225.1→162.1; and m/z 3-OH KYN-^15^N^13^C_2_ 228.1→163.1.

To analyze quinolinic acid, the analyte was first derivatized with methyl chloroformate under alkaline conditions. The chromatographic separation of the derivatized product was achieved using a Supelco, Ascentis Express, phenyl hexyl column, (2.7 µm particle size, 3.0 mm x 7.5 cm) maintained at 40°C. and elution achieved under isocratic conditions using 96% of mobile phase A comprised of ammonium formate (5 mM):acetonitrile (85:15; v:v) and mobile phase B comprised of 100% acetonitrile. The eluant was monitored using an API 5000 in ESI+ mode with transitions acquired for quinolinic acid 196.4→164.2 and quinolinic acid-D_3_ 199.4→167.2.

For the tryptophan assay, chromatographic separation was achieved using a Waters, Xbridge BEH C18 XP column (2.5 µm particle size, 2.1 x 50 mm) maintained at 40°C. A multistep gradient using mobile phase A comprised of water:formic acid (100:0.1, v:v) and mobile phase B comprised of acetonitrile:formic acid (100:0.1, v:v) was used for elution of tryptophan. A Sciex API 4000 in APCI+ mode was used to monitor transition ions for tryptophan 205.1→146.1 and tryptophan-D_5_ 210.1→150.0. For the 4-analyte assay, using a 200 µL sample, the lower limit of quantitation (LLOQ) was established at 1, 0.1, 0.1, and 0.25 ng/mL for kynurenine, Kynurenic acid , 3-OH-KYN, and anthranilic acid, respectively, in CSF and 10, 0.10, 1.0, and 0.25 ng/mL for kynurenine, Kynurenic acid , 3-OH-KYN, and anthranilic acid, respectively, in plasma. Based on analysis of a 25 µL sample, the LLOQ was 5 ng/mL in CSF and 500 ng/mL in plasma for tryptophan, and 1 ng/mL for quinolinic acid in both matrices.

In the 4-analyte assays in CSF and plasma, the acceptance criteria for each run were based on precision and accuracy values of ±20% bias (±25% at the LLOQ and LQC) and ≤20% RSD (≤25% at LLOQ and LQC), respectively, for the QCs for all four analytes. For the tryptophan and quinolinic acid assays, in both CSF and plasma, the acceptance criteria for precision and accuracy were set at ±15% bias and ≤15% RSD, respectively, at all QC levels except at LLOQ where ±20% bias and ≤20% RSD.

*Rationale for use of log transformations*

**Figure e1.**Descriptive histograms of the metabolite concentration distribution demonstrated that log-transformed data was more symmetrical (closer to “bell-shaped”) than the non-transformed data. Hence log transforms of the concentrations were used for subsequent statistical hypothesis tests.

*Abbreviations: 3-OH-KYN, 3-hydroxykynurenine; AA, anthranilic acid; KYN, kynurenine; KYNA, kynurenic acid; QA, quinolinic acid; TRP, tryptophan.*

**Figure e2: Scatter plots showing log transformed levels (ng/mL) of 3-OH-KYN, kynurenine, and quinolinic acid versus age.**

**Figure e3. Box plots for each of the metabolites of secondary interest showing log transformed concentrations (ng/mL) in control vs. PwHD.**

**
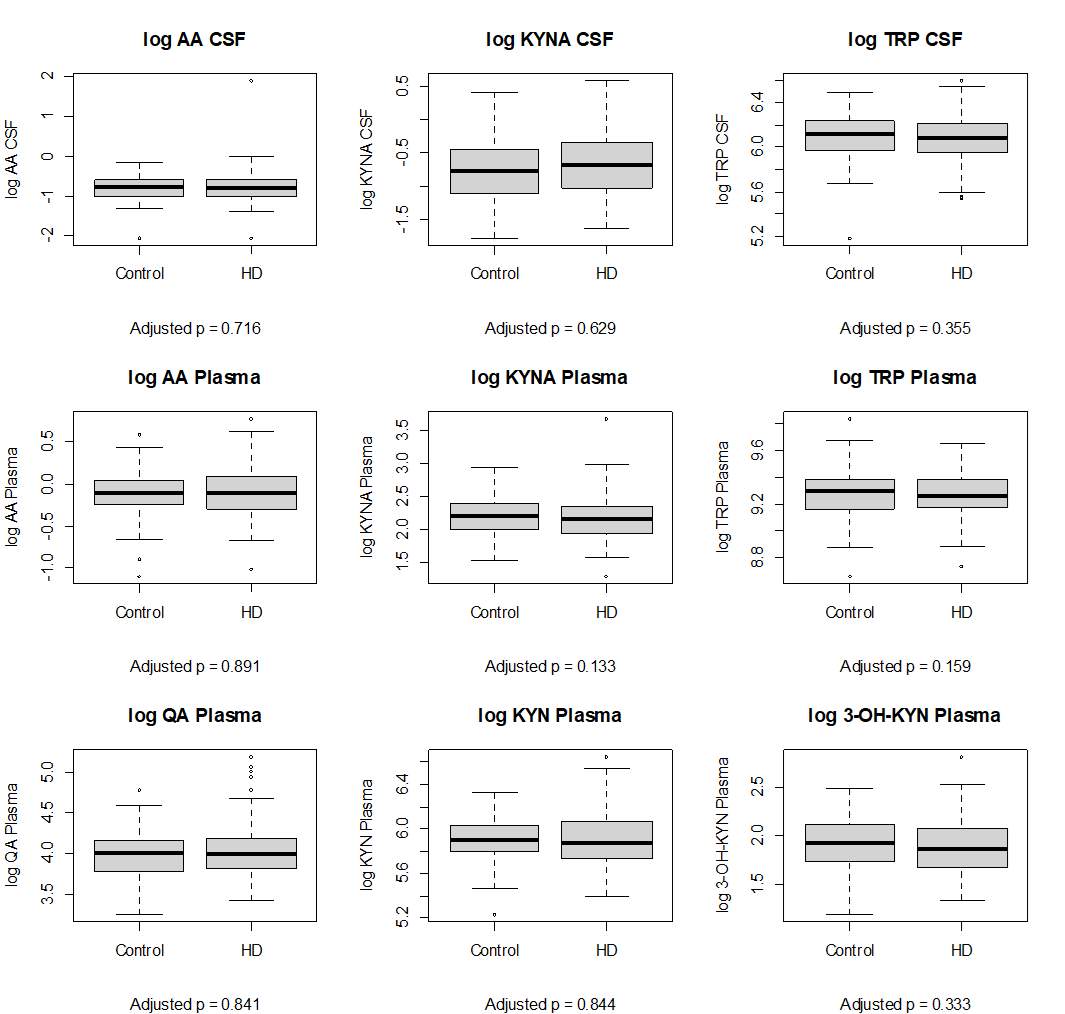
**

*Abbreviations: 3-OH-KYN, 3-hydroxykynurenine; AA, anthranilic acid; HD, Huntington’s disease; KYN, kynurenine; KYNA, kynurenic acid; QA, quinolinic acid; TRP, tryptophan.*

**Figure e3: Box plots for the KP metabolite ratios of interest showing log transformed concentrations (ng/mL) in control vs. PwHD**


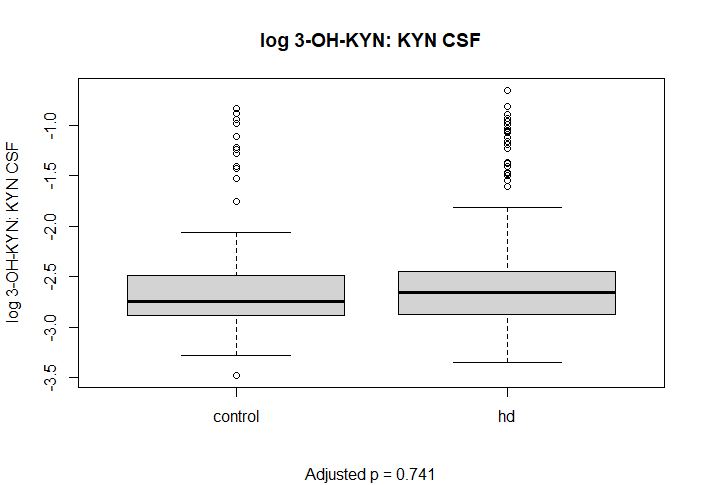

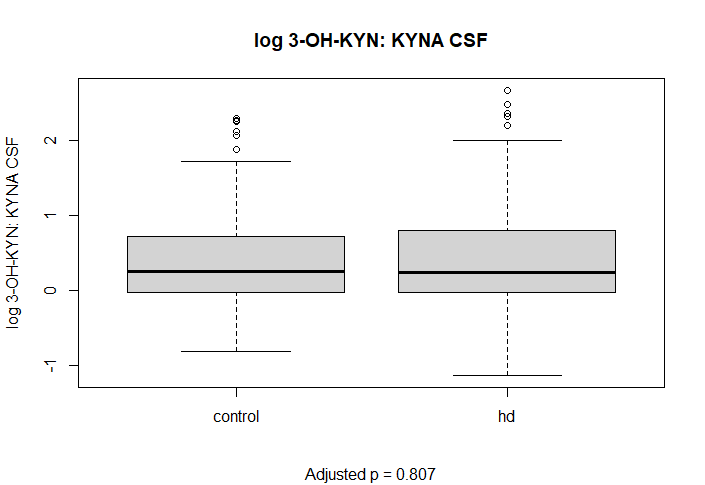


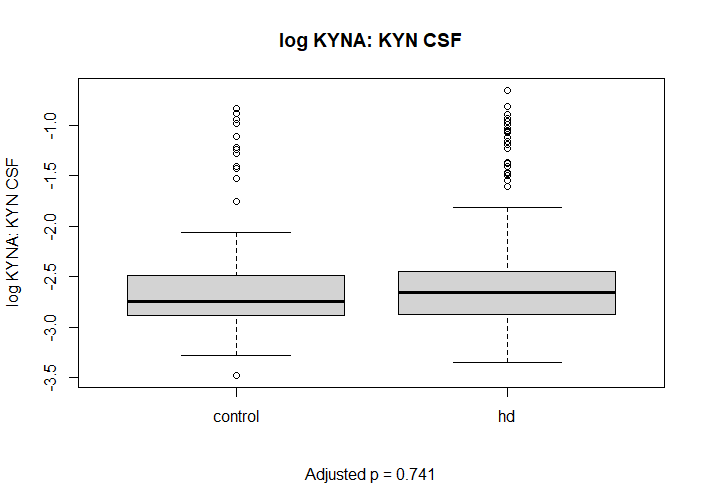

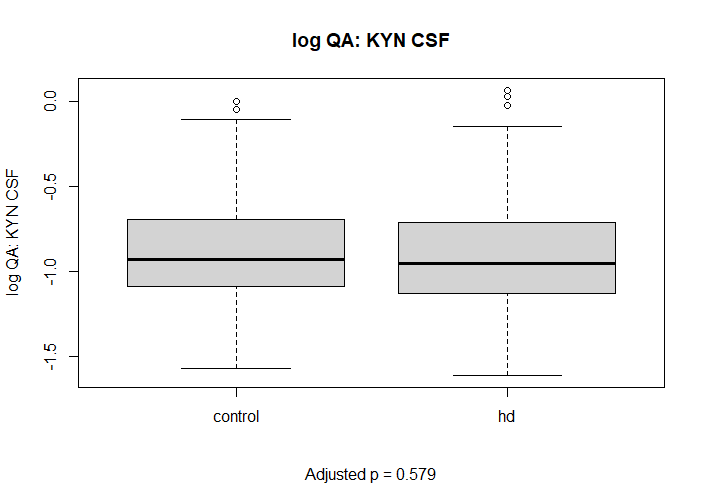


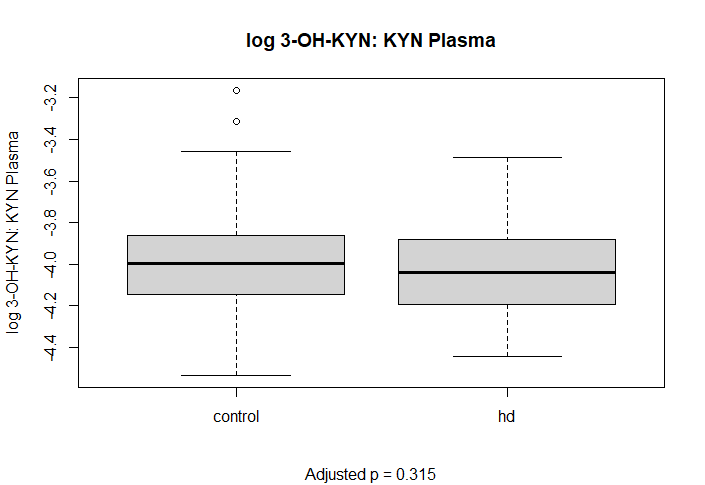

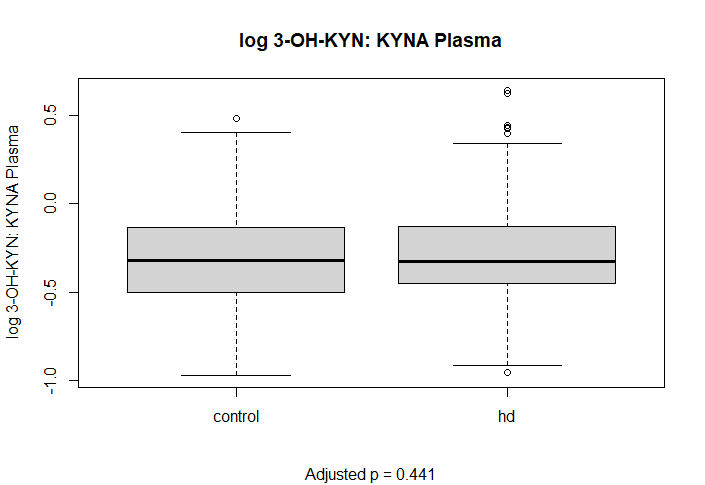


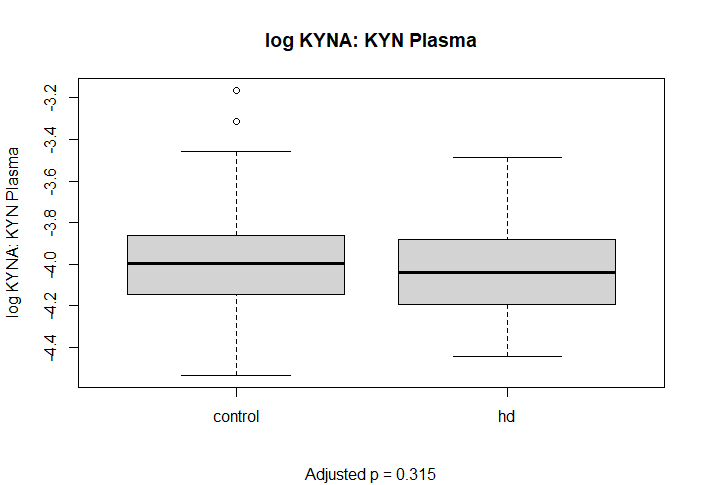

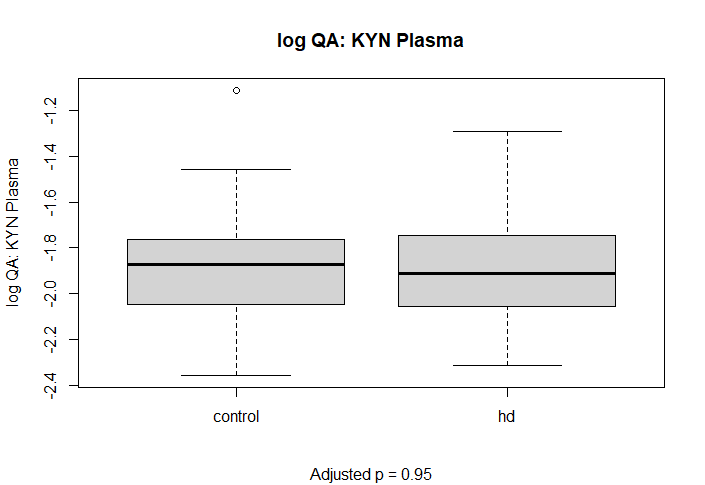


*Abbreviations: 3-OH-KYN, 3-hydroxykynurenine; AA, anthranilic acid; HD, Huntington’s disease; KYN, kynurenine; KYNA, kynurenic acid; QA, quinolinic acid; TRP, tryptophan.*

**Table e1. Unadjusted group comparisons (age and sex) of log concentrations (ng/mL) for the three primary outcome metabolites**

| **Metabolite** | **Parameter** | **Log Concentration**  **Difference (SE)** | **t value** | **p value** |
| --- | --- | --- | --- | --- |
| 3-OH-KYN | Age | 0.010(0.0039) | 2.49 | 0.014 |
|  | Male vs. Female | 0.011(0.0839) | 0.13 | 0.893 |
| Kynurenine | Age | 0.010(0.002) | 4.97 | 1.36E-06 |
|  | Male vs. Female | 0.013(0.045) | 0.30 | 0.767 |
| Quinolinic acid | Age | 0.014(0.002) | 6.19 | 3.04E-09 |
|  | Male vs. Female | 0.107(0.049) | 2.18 | 0.030 |

**Table e2. Metabolite principal component scores association with PwHD versus control status.**

|  | **Log OR (SE)** | **z value** | **p value** |
| --- | --- | --- | --- |
| PC1 score | 0.024 (0.148) | 0.160 | 0.873 |
| PC2 score | -0.040 (0.148) | -0.271 | 0.787 |
| PC3 score | -0.080 (0.147) | -0.544 | 0.586 |
| PC4 score | -0.024 (0.148) | -0.161 | 0.872 |
| PC5 score | -0.135 (0.146) | -0.922 | 0.357 |
| PC6 score | 0.256 (0.149) | 1.720 | 0.085 |

*log OR = change in log odds ratio of PwHD vs controls per principal component score standard deviation. The p value is 0.639 (deviance chi-square = 4.281) for the overall 6 df test of HD vs Controls using PC 1-6*

**Table e3. Association of KP log transformed metabolite levels in CSF (ng/mL) with clinical outcomes.**

| **Outcome** | **Predictor** | **Estimate** | **Std. Error** | **t value** | **p value** | ***fdr*** |
| --- | --- | --- | --- | --- | --- | --- |
| 3-OH-KYN | TMS | 0.003 | 0.003 | 0.949 | 0.344 | 0.669 |
|  | TFC | -0.030 | 0.020 | -1.533 | 0.128 | 0.399 |
|  | sdmt | -0.007 | 0.004 | -1.795 | 0.075 | 0.337 |
|  | swrt | -0.003 | 0.002 | -1.396 | 0.165 | 0.399 |
|  | scnt | -0.006 | 0.003 | -1.815 | 0.072 | 0.337 |
|  | cUHDRS10 | -0.033 | 0.017 | -1.913 | 0.058 | 0.337 |
| Kynurenine | TMS | -0.003 | 0.002 | -2.140 | 0.034 | 0.337 |
|  | TFC | -0.004 | 0.011 | -0.360 | 0.720 | 0.893 |
|  | sdmt | 0.007 | 0.002 | 3.010 | 0.002 | 0.085 |
|  | swrt | 0.002 | 0.001 | 1.491 | 0.138 | 0.399 |
|  | scnt | 0.004 | 0.002 | 2.549 | 0.012 | 0.215 |
|  | cUHDRS10 | 0.013 | 0.009 | 1.380 | 0.170 | 0.399 |
| Quinolinic acid | TMS | -0.001 | 0.002 | -0.617 | 0.538 | 0.755 |
|  | TFC | -0.013 | 0.012 | -1.078 | 0.283 | 0.599 |
|  | sdmt | 0.001 | 0.002 | 0.606 | 0.545 | 0.755 |
|  | swrt | -0.0002 | 0.001 | -0.205 | 0.838 | 0.914 |
|  | scnt | 0.001 | 0.002 | 0.721 | 0.472 | 0.709 |
|  | cUHDRS10 | -0.002 | 0.010 | -0.206 | 0.837 | 0.914 |
| Anthranilic acid | TMS | 0.0001 | 0.002 | 0.077 | 0.939 | 0.966 |
|  | TFC | -0.020 | 0.015 | -1.357 | 0.177 | 0.399 |
|  | sdmt | 0.003 | 0.003 | 0.897 | 0.372 | 0.669 |
|  | swrt | -0.003 | 0.002 | -1.448 | 0.150 | 0.399 |
|  | scnt | -0.001 | 0.002 | -0.314 | 0.754 | 0.905 |
|  | cUHDRS10 | -0.009 | 0.013 | -0.730 | 0.467 | 0.709 |
| Kynurenic acid | TMS | -0.00009 | 0.002 | -0.043 | 0.966 | 0.966 |
|  | tfc | -0.007 | 0.015 | -0.435 | 0.664 | 0.854 |
|  | sdmt | 0.004 | 0.003 | 1.370 | 0.173 | 0.399 |
|  | swrt | 0.004 | 0.002 | 2.065 | 0.041 | 0.337 |
|  | scnt | 0.005 | 0.002 | 1.969 | 0.051 | 0.337 |
|  | cUHDRS10 | 0.001 | 0.013 | 0.097 | 0.923 | 0.966 |
| Tryptophan | TMS | -0.0008 | 0.001 | -0.823 | 0.412 | 0.675 |
|  | tfc | 0.001 | 0.008 | 0.215 | 0.830 | 0.914 |
|  | sdmt | 0.002 | 0.001 | 1.543 | 0.125 | 0.399 |
|  | swrt | 0.0004 | 0.001 | 0.522 | 0.602 | 0.803 |
|  | scnt | 0.0009 | 0.001 | 0.823 | 0.412 | 0.675 |
|  | cUHDRS10 | 0.005 | 0.006 | 0.919 | 0.360 | 0.669 |

*Abbreviations: TMS= UHDRS motor score, TFC = UHDRS total functional capacity scale, SDMT= symbol digit modality test, SWRT = Stroop word-reading test, SCNT = Stroop color-naming test, cUHDRS10 = composite UHDRS score, fdr = false discovery rate.*

**Table e4. Association of KP log transformed metabolite levels (ng/mL) in plasma with clinical outcomes.**

| **Outcome** | **Predictor** | **Estimate** | **Std. Error** | **t value** | **p value** | ***fdr*** |
| --- | --- | --- | --- | --- | --- | --- |
| 3-OH-KYN | TMS | -0.0006 | 0.001 | -0.470 | 0.639 | 0.748 |
|  | tfc | -0.009 | 0.009 | -1.014 | 0.313 | 0.563 |
|  | sdmt | 0.002 | 0.002 | 1.226 | 0.222 | 0.473 |
|  | swrt | 0.0002 | 0.001 | 0.207 | 0.836 | 0.862 |
|  | scnt | 0.001 | 0.001 | 0.811 | 0.419 | 0.639 |
|  | cUHDRS10 | 0.002 | 0.008 | 0.205 | 0.838 | 0.862 |
| Kynurenine | TMS | -0.002 | 0.001 | -1.597 | 0.113 | 0.338 |
|  | tfc | -0.007 | 0.008 | -0.913 | 0.363 | 0.623 |
|  | sdmt | 0.004 | 0.002 | 2.371 | 0.019 | 0.195 |
|  | swrt | 0.001 | 0.001 | 1.267 | 0.207 | 0.473 |
|  | scnt | 0.003 | 0.001 | 2.227 | 0.028 | 0.195 |
|  | cUHDRS10 | 0.005 | 0.007 | 0.739 | 0.462 | 0.639 |
| quinolinic acid | TMS | -0.002 | 0.001 | -1.153 | 0.251 | 0.502 |
|  | tfc | -0.012 | 0.010 | -1.223 | 0.224 | 0.473 |
|  | sdmt | 0.004 | 0.002 | 2.014 | 0.046 | 0.207 |
|  | swrt | 0.001 | 0.001 | 0.638 | 0.525 | 0.664 |
|  | scnt | 0.002 | 0.002 | 1.488 | 0.139 | 0.358 |
|  | cUHDRS10 | 0.002 | 0.008 | 0.208 | 0.835 | 0.862 |
| Anthranilic acid | TMS | -0.0007 | 0.001 | -0.463 | 0.644 | 0.748 |
|  | tfc | -0.006 | 0.010 | -0.622 | 0.535 | 0.664 |
|  | sdmt | 0.002 | 0.002 | 0.842 | 0.401 | 0.639 |
|  | swrt | -0.001 | 0.001 | -0.694 | 0.489 | 0.652 |
|  | scnt | 0.001 | 0.002 | 0.773 | 0.441 | 0.639 |
|  | cUHDRS10 | 0.0004 | 0.008 | 0.045 | 0.964 | 0.964 |
| Kynurenic acid | TMS | -0.004 | 0.001 | -2.488 | 0.014 | 0.195 |
|  | tfc | 0.019 | 0.010 | 1.858 | 0.065 | 0.235 |
|  | sdmt | 0.005 | 0.002 | 2.161 | 0.032 | 0.195 |
|  | swrt | 0.003 | 0.001 | 2.044 | 0.043 | 0.207 |
|  | scnt | 0.004 | 0.002 | 2.351 | 0.020 | 0.195 |
|  | cUHDRS10 | 0.020 | 0.009 | 2.279 | 0.024 | 0.195 |
| tryptophan | TMS | -0.001 | 0.001 | -1.683 | 0.095 | 0.310 |
|  | tfc | 0.002 | 0.005 | 0.434 | 0.665 | 0.748 |
|  | sdmt | 0.001 | 0.001 | 1.093 | 0.276 | 0.524 |
|  | swrt | 0.001 | 0.001 | 1.926 | 0.056 | 0.225 |
|  | scnt | 0.001 | 0.001 | 1.520 | 0.131 | 0.358 |
|  | cUHDRS10 | 0.003 | 0.004 | 0.768 | 0.444 | 0.639 |

*Abbreviations: TMS= UHDRS motor score, TFC = UHDRS total functional capacity scale, SDMT= symbol digit modality test, SWRT = Stroop word-reading test, SCNT = Stroop color-naming test, cUHDRS10 = composite UHDRS score, fdr = false discovery rate.*

**Table e5. CAP30 (age*CAG-30) relationship with CSF and plasma metabolites.**

| **Matrix** | **Metabolite** | ***Res.Df*** | ***RSS*** | ***Df*** | ***Sum of Sq*** | ***F*** | ***p value*** | ***fdr*** |
| --- | --- | --- | --- | --- | --- | --- | --- | --- |
| CSF | 3-OH-KYN | 138 | 48.331 | 1 | 1.775 | 5.069 | 0.026 | 0.078 |
|  | Kynurenine | 138 | 15.917 | 1 | 0.066 | 0.571 | 0.451 | 0.541 |
|  | Quinolinic acid | 139 | 20.839 | 1 | 1.236 | 8.242 | 0.005 | 0.028 |
|  | Anthranilic acid | 139 | 30.530 | 1 | 0.188 | 0.856 | 0.356 | 0.535 |
|  | Kynurenic acid | 137 | 30.248 | 1 | 0.498 | 2.256 | 0.135 | 0.271 |
|  | Tryptophan | 139 | 5.750 | 1 | 0.003 | 0.077 | 0.782 | 0.782 |
| Plasma | 3-OH-KYN | 138 | 10.581 | 1 | 0.025 | 0.330 | 0.567 | 0.680 |
|  | Kynurenine | 138 | 8.073 | 1 | 0.004 | 0.073 | 0.787 | 0.787 |
|  | Quinolinic acid | 138 | 13.288 | 1 | 0.225 | 2.332 | 0.129 | 0.387 |
|  | Anthranilic acid | 138 | 12.538 | 1 | 0.050 | 0.552 | 0.459 | 0.680 |
|  | Kynurenic acid | 138 | 14.855 | 1 | 0.062 | 0.577 | 0.449 | 0.680 |
|  | Tryptophan | 138 | 3.619 | 1 | 0.143 | 5.453 | 0.021 | 0.126 |

*Abbreviations: Res DF = residual degrees of freedom, RSS = residual sum of squares, Df = test numerator degrees of freedom, Sum of Sq. = sum of squares “explained” jointly by the main effect of CAG length and the age-by-CAG length interaction, fdr = false discovery rate*

**Table e6. Effect of replacing CAP30 score with explicit predictor terms for age, CAG length, and age-by-CAG interaction**

| **Metabolite** | **Res.Df** | **RSS** | **Df** | **Sum of Sq** | **F** | **p value** | **fdr** |
| --- | --- | --- | --- | --- | --- | --- | --- |
| **Age-by-CAG Tests of Association with CSF Metabolites** | | | | | | | |
| Log 3-OH-KYN | 137 | 48.31 | 1 | 1.215 | 3.446 | 0.066 | 0.152 |
| Logkynurenine | 137 | 14.48 | 1 | 0.338 | 3.198 | 0.076 | 0.152 |
| Logquinolinic acid | 138 | 18.97 | 1 | 0.000 | 0.002 | 0.969 | 0.969 |
| loganthranilic acid | 138 | 29.46 | 1 | 0.106 | 0.497 | 0.482 | 0.578 |
| Logkynurenic acid | 136 | 27.91 | 1 | 0.217 | 1.059 | 0.305 | 0.458 |
| Logtryptophan | 138 | 5.50 | 1 | 0.133 | 3.338 | 0.070 | 0.152 |
| **Joint Association of CAG and Age-by-CAG with CSF Metabolites** | | | | | | | |
| Log 3-OH-KYN_CSF | 136 | 46.97 | 2 | 2.557 | 3.701 | 0.027 | 0.119 |
| Log kynurenine | 136 | 14.48 | 2 | 0.339 | 1.593 | 0.207 | 0.311 |
| Log quinolinic acid | 137 | 18.36 | 2 | 0.606 | 2.262 | 0.108 | 0.216 |
| Log anthranilic acid | 137 | 29.41 | 2 | 0.154 | 0.359 | 0.699 | 0.699 |
| Log kynurenic acid | 135 | 27.91 | 2 | 0.219 | 0.529 | 0.590 | 0.699 |
| Log tryptophan | 137 | 5.37 | 2 | 0.259 | 3.306 | 0.040 | 0.119 |
| **Age-by-CAG Tests of Association with Plasma Metabolites** | | | | | | | |
| Log 3-OH-KYN_Plasma | 137 | 10.58 | 1 | 0.027 | 0.343 | 0.559 | 0.671 |
| Log kynurenine | 137 | 7.95 | 1 | 0.077 | 1.322 | 0.252 | 0.463 |
| Log quinolinic acid | 137 | 12.12 | 1 | 0.104 | 1.179 | 0.279 | 0.463 |
| Log anthranilic acid | 137 | 11.98 | 1 | 0.091 | 1.045 | 0.308 | 0.463 |
| Log kynurenic acid | 137 | 14.69 | 1 | 0.005 | 0.045 | 0.832 | 0.832 |
| Log tryptophan | 137 | 3.55 | 1 | 0.213 | 8.217 | 0.005 | 0.029 |
| **Joint Association of CAG and Age-by-CAG with Plasma Metabolites** | | | | | | | |
| Log 3-OH-KYN | 136 | 10.38 | 2 | 0.226 | 1.478 | 0.232 | 0.531 |
| Log kynurenine | 136 | 7.87 | 2 | 0.155 | 1.339 | 0.266 | 0.531 |
| Log quinolinic acid | 136 | 12.06 | 2 | 0.168 | 0.946 | 0.391 | 0.586 |
| Log anthranilic acid | 136 | 11.95 | 2 | 0.118 | 0.674 | 0.512 | 0.614 |
| Log kynurenic acid | 136 | 14.66 | 2 | 0.026 | 0.123 | 0.885 | 0.885 |
| Log tryptophan | 136 | 3.54 | 2 | 0.225 | 4.331 | 0.015 | 0.090 |

*Abbreviations: Res DF = residual degrees of freedom, RSS = residual sum of squares, Df = test numerator degrees of freedom, Sum of Sq. = sum of squares “explained” by age-by-CAG length interaction, fdr = false discovery rate.*
